# The role of angiogenesis in the pathology of multiple sclerosis

**DOI:** 10.1186/s13221-014-0023-6

**Published:** 2014-11-28

**Authors:** Justin Lengfeld, Tyler Cutforth, Dritan Agalliu

**Affiliations:** Department of Developmental and Cell Biology, University of California at Irvine, Irvine, CA 92697-2300 USA

**Keywords:** Multiple sclerosis, Angiogenesis, Hypoxia, Experimental autoimmune encephalomyelitis, Blood–brain barrier, Hypoxia, VEGF, Endothelial cell

## Abstract

Angiogenesis, or the growth of new blood vessels from existing vasculature, is critical for the proper development of many organs. This process is inhibited and tightly regulated in adults, once endothelial cells have acquired organ-specific properties. Within the central nervous system (CNS), angiogenesis and acquisition of blood–brain barrier (BBB) properties by endothelial cells is essential for CNS function. However, the role of angiogenesis in CNS pathologies associated with impaired barrier function remains unclear. Although vessel abnormalities characterized by abnormal barrier function are well documented in multiple sclerosis (MS), a demyelinating disease of the CNS resulting from an immune cell attack on oligodendrocytes, histological analysis of human MS samples has shown that angiogenesis is prevalent in and around the demyelinating plaques. Experiments using an animal model that mimics several features of human MS, Experimental Autoimmune Encephalomyelitis (EAE), have confirmed these human pathological findings and shed new light on the contribution of pre-symptomatic angiogenesis to disease progression. The CNS-infiltrating inflammatory cells that are a hallmark of both MS and EAE secrete several factors that not only contribute to exacerbating the inflammatory process but also promote and stimulate angiogenesis. Moreover, chemical or biological inhibitors that directly or indirectly block angiogenesis provide clinical benefits for disease progression. While the precise mechanism of action for these inhibitors is unknown, preventing pathological angiogenesis during EAE progression holds great promise for developing effective treatment strategies for human MS.

## Introduction

Multiple sclerosis is a chronic inflammatory disease of the CNS, whose hallmarks include blood–brain barrier (BBB) breakdown as well as CNS inflammatory infiltration, demyelination and eventual axonal destruction. Approximately 2.3 million people worldwide suffer from MS and its debilitating symptoms, which range from numbness and tingling to more severe examples of partial or complete paralysis [1]. Experimental autoimmune encephalomyelitis (EAE), one of several animal models used to study MS, recapitulates many inflammatory and demyelinating characteristics of the disease [2,3]. In this paradigm, immunizing mice with myelin protein plus complete adjuvant results in a T cell-mediated disease displaying CNS inflammatory infiltration, BBB leakage and demyelination [2].

Angiogenesis, or the sprouting of new blood vessels from existing vasculature, is most prevalent in rodents and humans during development, and is generally inactive in adults except under certain regulated conditions such as wound healing and during female reproductive cycles [4]. Within the CNS, the process of angiogenesis is integrated with a series of programmed changes in endothelial cells, which culminates in the formation of a tight barrier [5]. The key features of this barrier include tight junctions, low levels of transcytosis and transporters for specific molecules, which together ensure a selectively permeable barrier that maintains CNS homeostasis and protects the tissue from intrusion by unwanted molecules, ions and cells [6,7]. Several cell types and signaling pathways that regulate developmental CNS angiogenesis and BBB formation have been described [8-12]. While there is increasing evidence that angiogenesis occurs in CNS diseases with impaired barrier function such as stroke [13] or MS [14,15], the role of angiogenesis in human MS pathology remains unclear. Here we highlight in a brief review recent studies suggesting that angiogenesis, stimulated by the presence of invading immune cells, plays a role in both the progression and severity of human MS.

### Timing of angiogenesis in the EAE model and its relevance to recovery

Blood vessel abnormalities such as impaired barrier function have long been associated with MS lesions [16]; however only recently has there been evidence confirming both the presence of angiogenesis in MS patients as well as establishing its onset in relation to disease progression. Angiogenesis, as measured by an increase in vessel number and size, was first described to be present not only within and at the edge of acute MS lesions but also in the area surrounding the plaque, where it is often associated with areas of inflammation [15]. In further support of these findings, Proescholdt *et al.* observed blood vessels within lesions that are composed of reactive endothelial cells displaying an irregular morphology, consistent with angiogenesis [17]. Markers for proliferating endothelial cells, such as endoglin, are also significantly more prominent in MS patient samples as compared to controls [18].

Although pathological studies in human MS tissue have demonstrated that angiogenesis occurs in MS lesions [14], determining when this process begins in patients has been challenging. Recently, magnetic resonance imaging (MRI) studies on cerebral perfusion differences in MS patients have begun to shed light on when angiogenesis begins in relation to disease progression. This method has proven effective in assessing whether angiogenesis is present, as established by correlation between increased cerebral perfusion and histologically determined vessel density [19]. Using MRI to examine cerebral perfusion of gadolinium-enhancing lesions, Wuerfel *et al.* found that both local blood flow and blood volume are significantly increased, indicating an increase in blood vessel density [20]. Furthermore, there is evidence that increased cerebral perfusion precedes detection of lesions with gadolinium by up to 3 weeks [20,21]. These studies indicate that angiogenesis takes place early during MS progression, even before formation of lesions with impaired endothelial barrier function.

In the EAE mouse model for MS, angiogenesis has been confirmed histologically in many areas with clear inflammation and demyelinating lesions [22-25]. Boroujerdi *et al.* have reported that the total area of CD31^+^ vessels in the spinal cord increases significantly, as early as 7 days post-induction of EAE in mice, which is a week before clinical symptoms appear [25]. In support of such pre-symptomatic increases in blood vessel density, Seabrook *et al.* have reported increased blood vessel density during the relapse phase (27 days post-induction) of EAE in rats [22]. Although there are clear differences between the two animal models and species, the role of angiogenesis is increasingly being considered as a contributing factor to MS pathology.

While the timing of angiogenesis during disease progression in MS and EAE is still under investigation, its presence in both forms of the disease raises the question whether angiogenesis is beneficial or detrimental to clinical recovery. The majority of current studies would suggest that angiogenesis is detrimental to MS pathogenesis [18,23,24,26]. The pre-symptomatic increase in angiogenesis observed by Boroujerdi *et al.* supports a pathogenic role for this process. Further corroborating these findings is the observation that blocking the strong angiogenic stimulator vascular endothelial growth factor (VEGF) during EAE progression results in an improved clinical score and attenuation of both demyelination and inflammation [24,27]. In contrast, Dore-Duffy and colleagues report that pre-conditioning mice under mild hypoxia, in order to promote angiogenesis, reduces the clinical severity of EAE as well as inflammatory infiltration into the CNS [28]. Furthermore, new blood vessels formed during EAE progression secrete prostacyclin, a trophic factor that enhances axonal remodeling and functional recovery [29]. These opposing findings demonstrate the complex role that angiogenesis may play in MS and EAE. Further studies will be needed to fully unravel the ramifications of new blood vessel growth for disease progression.

### Are inflammatory cell infiltration and angiogenesis linked?

Several factors contribute to angiogenesis in EAE and MS lesions; it is known that a state of hypoxia exists in active MS lesions. Such hypoxic regions are due to numerous changes in the local lesion environment. Lassmann *et al.* [30] showed that active MS lesions exhibit a marked expression of hypoxia-inducible factor 1-alpha (HIF-1α). Demyelinated axons attempt to compensate for loss of efficient saltatory action potential propagation by increasing expression of leaky Na^+^ channels, which further exacerbates the energy demands of the tissue [31]. Active MS lesions also display elevated levels of inducible nitric oxide (NO) synthase, resulting in high levels of NO [32] that inhibit mitochondrial respiration [33]. The increased energy needs of demyelinated axons and the influx of immune cells, along with locally secreted factors, combine to create an environment where demand outpaces supply, thus engendering a hypoxic condition that is conducive to angiogenesis.

**Figure 1:**
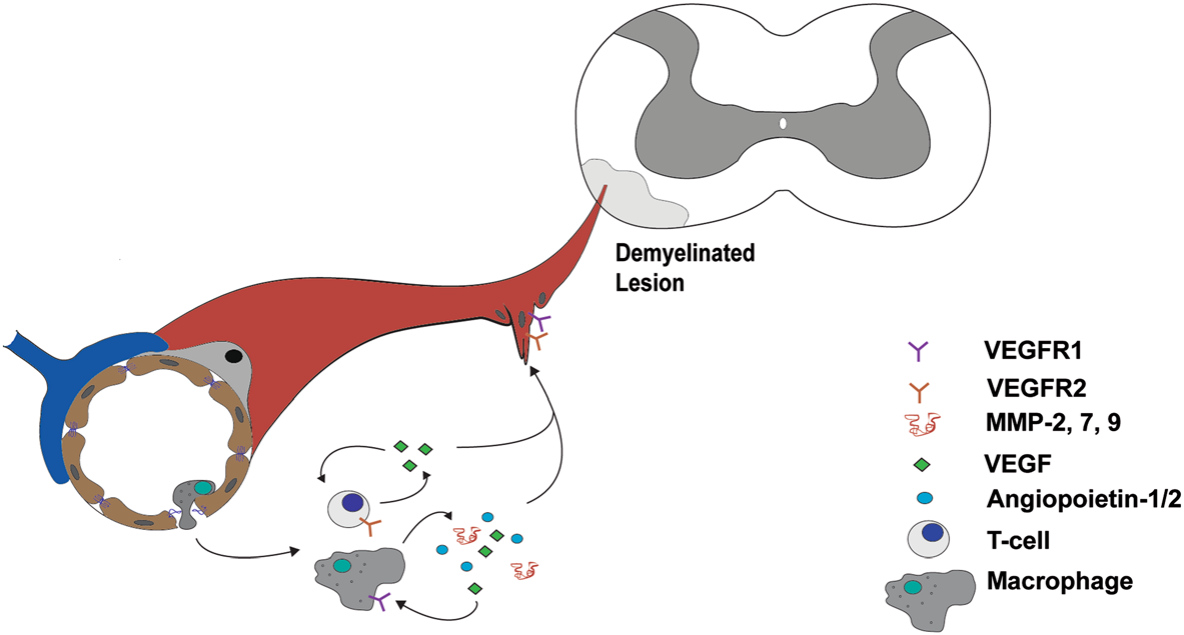
**Inflammatory cell infiltration is linked to angiogenesis in both MS and EAE.** Inflammatory cells (T cells and macrophages) infiltrate the central nervous system parenchyma of a demyelinated lesion in both human MS or mouse EAE (depicted in the schematic diagram). After entering the hypoxic environment of the lesion, T cells and macrophages secrete pro-angiogenic factors (VEGF, angiopoietin1/2, and MMP-2, −7, −9) that both promote angiogenesis and exacerbate lesion pathology. These factors act both in a paracrine manner in endothelial cells to stimulate angiogenesis, as well as in an autocrine fashion, to exacerbate the inflammatory response of both T cells and macrophages.

Hypoxic conditions within the CNS also modulate immune cell responses. Activated T cells that cross the BBB into this locally hypoxic CNS environment shift their profile of cytokine expression. Mor *et al.* found that hypoxia induces vascular endothelial growth factor (VEGF) and vascular endothelial growth factor receptor 2 (VEGFR2) mRNA and protein expression in T cells [34]. The cytokine signature of T cells exposed to VEGF shifts to a Th1 pro-inflammatory profile, with increased interferon gamma (INFγ) secretion and decreased interleukin-2 (IL-2) production [34]. When adoptive EAE transfer was then induced by injecting T cells that had been pre-treated with VEGF into rats, this resulted in an earlier onset and a more severe, prolonged disease [34]. Thus, activated T cells contribute to nurturing angiogenesis by secreting VEGF into a hypoxic environment, which may act in an autocrine fashion to exacerbate the inflammatory response (Figure 1).

Macrophages make up a large component of the inflammatory infiltrate in MS and EAE [35]. The phenotype of macrophage subtypes that develop during EAE is largely influenced by both exposure to T cells that are present in the lesion and the local hypoxic/inflammatory environment. INFγ, the predominant cytokine of inflammatory Th1 cells, shifts macrophages into a pro-inflammatory M1 phenotype [36]. M1 macrophages are predominantly found in active inflammatory MS lesions and the early stages of EAE in rats [37,38]. However, the fraction of macrophages that exhibit M2 markers is increased at both the peak stage of the disease as well as during the remission phase, at least in the relapsing-remitting EAE model in rats [37,38]. The complexity in phenotype switching that occurs in macrophages subtypes during MS lesion development and EAE disease underscores the complex and dynamic environment that these cells are exposed to as they cross the damaged BBB during disease progression [39]. Anti-inflammatory M2 macrophages, and not type M1, have been shown to have angiogenesis-promoting properties *in vivo* and *in vitro*, mainly through secretion of FGF [36] that correlates both with the timing of their maturation in the lesions and activation of angiogenesis. Infiltrating macrophages secrete factors that not only contribute to disease pathogenesis but also promote angiogenesis [40]. For example, elevated matrix metalloproteinase (MMP) levels, specifically MMP-2, −7 and −9, have been observed in both human MS patients and rodent EAE models [41-43]. MMPs, including macrophage-secreted MMP-7 and-9 [40,44], are extracellular zymogens that remain inactive until being cleaved by plasminogen activators. Once activated, MMPs digest components of the extracellular matrix (ECM), facilitating immune cell infiltration into the CNS in the context of MS and EAE [45] as well as creating a path through the basement membrane for endothelial cell migration during angiogenesis [46] (Figure 1).

In addition to contributing to ECM modifications and facilitating angiogenesis, macrophages also produce several angiogenic factors including VEGF, platelet-derived growth factor (PDGF) and angiopoietin-1 and - 2 [47] (Figure 1). The angiogenic contribution of macrophages is not limited to direct secretion, however, because they also play a role in freeing the reservoir of VEGF that is bound to the ECM by secreting MMPs that degrade the matrix and liberate bound VEGF [48]. Macrophages also express VEGFR-1, and can respond in a paracrine fashion to VEGF that they either produce or liberate from the ECM, by migrating towards angiogenic environments [47]. Consequently, the role played by infiltrating macrophages in MS and EAE is multifaceted, with overlapping functions contributing to both angiogenic and inflammatory responses (Figure 1).

Several compelling studies have investigated the effects of angiogenesis blockade on EAE progression and inflammation. Bevacizumab, a monoclonal antibody that binds VEGF and prevents interaction with its receptor [49], significantly decreases both EAE clinical scores and the number of CD4^+^ T cells in the CNS [27] when administered to mice on the day they began to show clinical symptoms. Similar results have been observed using the VEGFR2 receptor inhibitor SU5416, with mice showing significantly reduced acute EAE clinical scores and inflammatory infiltration, as well as fewer blood vessels per tissue section, when compared to untreated EAE mice [24]. Use of a second monoclonal VEGF-binding antibody, B20-4.1.1 [50], has recently shed light on the mechanism by which inhibiting angiogenesis may ameliorate EAE. B20-4.1.1 significantly reduced angiogenesis in the CNS, vascular permeability and clinical EAE scores during disease progression, without the concomitant reduction in CNS T cell infiltration seen using Bevacizumab or SU5416 [51]. Nevertheless, B20-4.1.1 diminished peripheral T cell activation, which was attributed by the authors to their observed reduction in EAE scores. Therefore, this study would suggest that CNS angiogenesis is linked to peripheral T cell activation rather than CNS accumulation, and provides a more complex perspective on possible secondary effects that inhibition of VEGF signaling has for CNS vasculature.

Whereas indirect inhibition of angiogenesis, by blocking angiogenic factors such as VEGF, shows promise for attenuating disease progression in EAE [24,27,51], direct inhibition also has clinical benefits in the EAE animal model [51]. Direct inhibition of angiogenesis by administering K(1–3) [50], a compound that contains the first 3 kringle domains from angiostatin and acts directly on vascular endothelial cells to inhibit angiogenesis [52], reduces both EAE clinical scores and IL-17 production by peripheral T cells without diminishing CNS infiltration [51]. While the precise mechanism of action underlying this phenomenon is unknown, it is clear that altering levels of angiogenesis is a potentially promising therapeutic approach for treating MS.

### Potential signals regulating angiogenesis

Since a hypoxia-like state is an important component of multiple sclerosis pathology [30,31], factors that function as angiogenic signals in either ischemic diseases (e.g. stroke) or tumor-induced angiogenesis may also promote angiogenesis in both MS and EAE [53,54]. High levels of hypoxia-inducible factor 1 (HIF-1), a transcription factor essential for VEGF-induced angiogenesis, have been found in active MS lesions [26,30,55,56]. Similar expression patterns for VEGF have been reported, with many VEGF-expressing cells observed in or adjacent to active lesions for both MS and EAE [17,22]. Furthermore, endothelin- 1 [57] and angiopoietin-2 [27] have both been shown to enhance the angiogenic effects of VEGF, and are significantly elevated in serum from MS patients [27,58]. The abundant evidence for both VEGF and VEGF-enhancing factor expression demonstrates a key role for this pathway in CNS angiogenesis during MS progression. NO is a ubiquitous molecule with numerous functions, including vasodilation and neurotransmission. It has been shown that NO contributes both directly and indirectly to neo-angiogenesis in inflammatory diseases [59]. Giovannoni *et al.* [60] have shown that NO levels are elevated in MS, and correlate with both serum inflammation and MRI markers of MS disease progression. Fibroblast growth factor (FGF) has also been implicated in contributing to angiogenesis [61]. Finally, gene microarray analysis of MS lesions has shown that transcript levels of FGF-12 and FGF-2 homolog are elevated 10.7- and 4.7-fold, respectively [62], and MS patients exhibit significantly elevated serum levels of FGF [63].

In addition to hypoxia-associated factors, many molecules that play a role in the pathology of MS also have angiogenesis-modulating characteristics. Tumor necrosis factor-alpha (TNFα) and INFγ, two prominent inflammatory cytokines that are present in EAE and MS [64], can promote angiogenesis [65]. Moreover, some MMPs (−1, −2 and −9) promote invasion of inflammatory cells into the CNS during MS progression [66], and contribute to proteolytic degradation of the extracellular matrix to allow angiogenic sprouting [67]. Further studies are needed to evaluate any *bona fide* effects these factors may have on neo-angiogenesis versus inflammation in the context of MS pathology.

## Conclusions

Much progress has been made in determining the contribution that angiogenesis makes during MS disease progression. Using the EAE animal model and human MS tissue samples, it is increasingly clear that neo-angiogenesis is stimulated during disease progression [15,17,18,22-24,27]. Furthermore, pharmacological inhibition of angiogenesis with various compounds suggests that it is beneficial for disease outcome [24,27,51]. Continued investigation into the potential signals that modulate the angiogenic response has already yielded therapeutic targets for treatment, and may provide additional candidates. While the mechanisms used by current anti-angiogenic compounds have yet to be elucidated, their efficacy supports ongoing research into their potential as therapeutic agents.

## Abbreviations

BBB: Blood–brain barrier
CNS: Central nervous system
EAE: Experimental autoimmune encephalomyelitis
FGF: Fibroblast growth factor
HIF: Hypoxia-inducible factor
INFγ: Interferon gamma
MMP: Matrix metalloproteinase
MRI: Magnetic resonance imaging
MS: Multiple sclerosis
NO: Nitric oxide
PDGF: Platelet-derived growth factor
TNFα: Tumor necrosis factor-alpha
VEGF: Vascular endothelial growth factor
VEGFR2: Vascular endothelial growth factor receptor 2
